# Analysis of Vaccine Reactions After COVID-19 Vaccine Booster Doses Among Pregnant and Lactating Individuals

**DOI:** 10.1001/jamanetworkopen.2022.30495

**Published:** 2022-09-08

**Authors:** Alisa Kachikis, Janet A. Englund, Isabela Covelli, Yael Frank, Candace Haghighi, Michael Singleton, Alison L. Drake, Linda O. Eckert

**Affiliations:** 1 Department of Obstetrics and Gynecology, University of Washington, Seattle; 2 Seattle Children’s Hospital Research Institute, Department of Pediatrics, University of Washington, Seattle; 3 School of Medicine, University of Washington, Seattle; 4 School of Medicine, Tel Aviv University, Tel Aviv, Israel; 5 School of Medicine, Wake Forest University, Winston-Salem, North Carolina; 6 Institute of Translational Health Sciences, University of Washington, Seattle; 7 Department of Global Health, University of Washington, Seattle

## Abstract

**Importance:**

COVID-19 vaccine boosters or third doses are recommended for adolescents and adults who completed their initial COVID-19 vaccine course more than 5 months prior. Minimal data are available on COVID-19 vaccine booster or third dose reactogenicity among pregnant and lactating individuals.

**Objective:**

To describe the reactions to the booster or third dose of the COVID-19 vaccine and vaccine experiences among pregnant and lactating individuals.

**Design, Setting, and Participants:**

Beginning in October 2021, a follow-up Research Electronic Data Capture (REDCap) survey regarding a COVID-19 vaccine booster or third dose was sent to 17 504 participants in an ongoing online prospective cohort study on COVID-19 vaccines among pregnant and lactating individuals. A convenience sample of adults enrolled in the online prospective study who were pregnant, lactating, or neither pregnant nor lactating at the time of their booster or third dose was eligible for this follow-up survey; 17 014 (97.2%) completed the follow-up survey.

**Exposure:**

Receipt of a booster or third dose of the COVID-19 vaccine.

**Main Outcomes and Measures:**

Self-reported vaccine reactions less than 24 hours after the dose.

**Results:**

As of April 4, 2022, 17 014 eligible participants (mean [SD] age, 33.3 [3.5] years) responded to the booster or third dose survey; of these, 2009 (11.8%) were pregnant at the time of their booster or third dose, 10 279 (60.4%) were lactating, and 4726 (27.8%) were neither pregnant nor lactating. After a COVID-19 booster or third dose, most individuals (14 074 of 17 005 [82.8%]) reported a local reaction, and 11 542 of 17 005 (67.9%) reported at least 1 systemic symptom. Compared with individuals who were neither pregnant nor lactating, pregnant participants were more likely to report any local reaction to a COVID-19 booster or third dose (adjusted odds ratio [aOR], 1.2; 95% CI, 1.0-1.4; *P* = .01) but less likely to report any systemic reaction (aOR, 0.7; 95% CI, 0.6-0.8; *P* < .001). Most pregnant (1961 of 2009 [97.6%]) and lactating (9866 of 10 277 [96.0%]) individuals reported no obstetric or lactation concerns after vaccination.

**Conclusions and Relevance:**

This study suggests that COVID-19 vaccine boosters or third doses were well tolerated among pregnant and lactating individuals. Data to evaluate tolerability of boosters or additional doses among pregnant and lactating individuals will be important as they are considered for these populations.

## Key Points

**Question:**

What experiences have pregnant and lactating individuals had after the COVID-19 vaccine booster or third dose?

**Findings:**

This cohort study of 17 014 participants found that most individuals (82.8%) reported a local reaction and that 67.9% reported at least 1 systemic symptom after a COVID-19 vaccine booster or third dose. Most pregnant (97.6%) and lactating (96.0%) individuals reported no obstetric or lactation concerns after vaccination.

**Meaning:**

This study suggests that the COVID-19 vaccine booster or third dose was well tolerated among pregnant and lactating individuals.

## Teaser

This cohort study describes the reactions to boosters or third doses of the COVID-19 vaccine and vaccine experiences among pregnant and lactating individuals.

## Introduction

COVID-19 vaccine boosters or additional doses are recommended for persons who completed their initial COVID-19 vaccine course more than 5 months after receipt of the Pfizer-BioNTech BNT162b2 or Moderna mRNA-1273 primary vaccine series or more than 2 months after receipt of the Janssen JNJ-78436735 vaccine.^1^ It is also recommended that individuals who are immunocompromised receive an additional (or third) dose after the initial vaccine series.^1^ In addition, a second booster dose is now recommended for US adults older than 50 years or who are immunocompromised or have certain health conditions.^1^ COVID-19 vaccines and, particularly, receipt of a booster or third dose after completion of the primary vaccine series have been shown to significantly decrease risk of infection, urgent care or emergency department encounters, hospitalizations, and death among adults, particularly in the context of SARS-CoV-2 variants.^2,3^ In clinical trials, booster doses of mRNA or adenovirus vector vaccines were well tolerated, with similar or lower rates of local or systemic reactions to the last dose in the primary series.^4,5,6^ In addition, booster doses of the BNT162b2 (Pfizer-BioNTech) COVID-19 vaccine have been shown to be safe among 12- to 17-year-old adolescents, with only rarely reported serious adverse events.^7^ Although studies have shown the COVID-19 primary vaccine series to be well tolerated and safe for pregnant and lactating individuals,^8,9^ data are lacking on COVID-19 vaccine boosters or third doses among this population. We investigated reactions to the COVID-19 vaccine booster doses and vaccine experiences among pregnant and lactating individuals.

## Methods

We launched an online prospective cohort study in January 2021 of adults who were pregnant, lactating, and/or planning pregnancy at the time of COVID-19 vaccination. This study was determined to be exempt from institutional review board review under Common Rule category 2 by the University of Washington Human Subjects Division. Individuals were recruited and enrolled online in the University of Washington COVID-19 Vaccine in Pregnancy and Lactation Registry, which was distributed via chain referral or snowball sampling initially on health care professional social media pages and listservs and then more broadly across social media sites using Research Electronic Data Capture (REDCap), version 12.1.1 (Vanderbilt University). Participants in the registry were then asked to participate in an ongoing prospective survey–based study after written informed consent was obtained, as previously described.^8^ Beginning in October 2021, an additional survey on reactions to the booster or third dose of the COVID-19 vaccine was distributed to enrolled participants who stated that they had received a booster or third dose during the follow-up and were asked to report their reactions and experiences. In the survey (the eAppendix in the Supplement provides for additional information regarding survey questions), participants were asked to report on local reactions (injection site pain, erythema, and/or swelling) or systemic reactions (myalgia, fatigue, gastrointestinal symptoms, fever, chills, anaphylactic reactions, or rash or hives) after the booster or third dose. Pregnant and lactating participants were asked to report pregnancy-related concerns (contractions, “breaking bag of water,” low amniotic fluid level, concerning baby heart monitoring, concerns about baby movement, vaginal bleeding, higher blood pressures, higher blood glucose levels, and/or more vaginal discharge) or lactation-related concerns (changes in breast milk supply and/or concerns about the infant). Information was also collected regarding the vaccine dose, work or activities of daily living, motivations to receive the booster dose, and sources for information about the booster dose. Race and ethnicity data were collected to report diversity representation within our study population. Categorization of race and ethnicity followed the Centers for Disease Control and Prevention’s National Health Interview Survey race and ethnicity categories^10^; participants could also select an “other” designation as an additional category, with an opportunity to specify the other race(s). Health care professionals included obstetrics, primary, or other health care providers. We followed the Strengthening the Reporting of Observational Studies in Epidemiology (STROBE) reporting guideline.^11^

Univariable comparisons of baseline characteristics for pregnant, lactating, and neither pregnant nor lactating individuals were performed using the χ^2^ test for categorical variables or 1-way analysis of variance for continuous variables. Separate multivariable logistic regression models were constructed to compare vaccine reactions and experiences between individuals who were pregnant with those who were neither pregnant nor lactating at the time of their booster or third dose and to compare these outcomes between individuals who were lactating with individuals who were neither pregnant nor lactating at the time of their booster or third dose. All multivariable models were adjusted for group differences with respect to vaccine type, age, race, area of employment, educational level, parity, and number of days elapsed from receiving the vaccine dose until completing the survey. For the outcomes “number of local reactions” and “number of systemic reactions,” multivariable analyses were performed in a similar manner using linear regression. Statistical analyses were conducted using Stata, version 14.2 (StataCorp LLC). All *P* values were from 2-sided tests and results were deemed statistically significant at *P* < .05.

## Results

As of April 4, 2022, 17 014 of 17 504 eligible participants (mean [SD] age, 3.3 [3.5] years) who reported they had received a COVID-19 vaccine booster or third dose completed the survey for this vaccine dose, most of whom (16 413 of 16 908 [97.1%]) resided in the United States and in all 50 states and several US territories, including Guam, Northern Mariana Islands, Puerto Rico, and US Virgin Islands; self-identified as White (15 576 of 16 903 [92.1%]), self-identified exclusively as White (15 068 of 17 005 [88.6%]), self-identified as non-Hispanic (15 958 of 16 892 [94.5%]), and self-identified as female (16 856 of 16 909 [99.7%]); had at least a college education (16 249 of 16 902 [96.1%]); and were employed in health care (9175 of 16 371 [56.0%]) (Table 1). Of these 17 014 participants, 2009 (11.8%) were pregnant at the time of their booster or third dose, 10 279 (60.4%) were lactating, and 4726 (27.8%) were neither pregnant nor lactating (including participants who were, at the time, no longer pregnant or lactating or who were planning a pregnancy). Among pregnant participants, receipt of a booster or third dose was similar by trimester: 530 of 2008 participants (26.4%) in the first trimester, 733 of 2008 participants (36.5%) in the second trimester, and 745 of 2008 participants (37.1%) in the third trimester. Among 16 989 individuals who reported their vaccine type for their booster or third dose, most received the BNT162b2 (10 319 [60.7%]) or mRNA-1273 (6651 [39.2%]) vaccines. The most common reason cited for receiving a booster or third dose of a COVID-19 vaccine was working in a health care field (8696 of 17 014 [51.1%]).

**Table 1.** Baseline Participant Characteristics (N = 17 014)^a^

Characteristic	Pregnant (n = 2009)		Lactating (n = 10 279)		Neither pregnant nor lactating (n = 4726)^b^		*P* value^c^
	No.	No. (%)	No.	No. (%)	No.	No. (%)	
Type of vaccine for booster or third dose	2007		10 264		4718		
BNT162b2		1311 (65.3)		6212 (60.5)		2796 (59.3)	<.001
mRNA-1273		691 (34.4)		4043 (39.4)		1917 (40.6)	
JNJ-78436735		5 (0.3)		9 (0.1)		5 (0.1)	
Booster same type as primary vaccine series	1999	1782 (89.1)	10 217	8723 (85.4)	4686	4023 (85.9)	<.001
Time from completion of primary vaccine series to booster or third dose, mean (SD), d	1992	253.0 (44.1)	10 179	251.2 (36.3)	4670	259.8 (37.2)	<.001
Time from vaccine booster or third dose to survey, mean (SD), d	2007	45.2 (41.3)	10 270	46.9 (45.5)	4722	53.4 (47.7)	<.001
Age, mean (SD), y	2009	32.6 (3.2)	10 279	33.5 (3.6)	4726	33.4 (3.5)	<.001
Gravidity, mean (SD)^d^	1985	1.6 (1.2)	10 115	2.2 (1.3)	4652	2.0 (1.3)	<.001
Parity, mean (SD)^d^	1986	0.9 (0.7)	10 131	1.1 (1.0)	4664	1.3 (0.9)	
Trimester of pregnancy	2008		NA		NA		<.001
First		530 (26.4)		NA		NA	
Second		733 (36.5)		NA		NA	
Third		745 (37.1)		NA		NA	
Race^e^	1998		10 207		4698		
American Indian or Alaska Native		7 (0.4)		93 (0.9)		28 (0.6)	.01
Asian		125 (6.3)		790 (7.7)		312 (6.6)	.01
Black or African American		21 (1.1)		140 (1.4)		59 (1.3)	.49
Native Hawaiian or Other Pacific Islander		7 (0.4)		45 (0.4)		21 (0.4)	.84
White		1881 (94.1)		9340 (91.5)		4355 (92.7)	<.001
Other		19 (1.0)		139 (1.4)		48 (1.0)	.11
Hispanic ethnicity	1997	110 (5.5)	10 200	578 (5.7)	4695	246 (5.2)	.57
Education	2001		10 209		4692		
Some college or less		66 (3.3)		380 (3.7)		207 (4.4)	<.001
Bachelor’s degree (eg, BA, BS)		617 (30.8)		2873 (28.1)		1448 (30.9)	
Master’s degree		778 (38.9)		3674 (36.0)		1687 (36.0)	
Doctorate or professional degree		540 (27.0)		3282 (32.2)		1350 (28.8)	
Area of employment	1935		9877		4559		
Health care		1073 (55.5)		5435 (55.0)		2667 (58.5)	.01
Academics or science		234 (12.1)		1221 (12.4)		544 (11.9)	
Teacher or childcare		116 (6.0)		659 (6.7)		279 (6.1)	
Office work or technology		222 (11.5)		1056 (10.7)		452 (9.9)	
Other^f^		290 (15.0)		1506 (15.3)		617 (13.5)	

Abbreviation: NA, not applicable.

^a^ All variables are based on pregnancy status at the time of COVID-19 vaccine booster or third dose.

^b^ Participants in this cohort were neither pregnant nor lactating at the time of the COVID-19 booster or third dose.

^c^ The χ^2^ test for categorical variables or 1-way analysis of variance for continuous variables.

^d^ Gravidity and parity as reported at initial enrollment.

^e^ Not mutually exclusive. Options for race were outlined following the Centers for Disease Control and Prevention’s National Health Interview survey race categories.^10^ Participants could select “other” if they did not select outlined race categories or in addition to other race categories and were given the opportunity to specify their own category. Missing numbers indicate participants who selected “Prefer not to answer.”

^f^ Includes military personnel, first responders, persons working in agriculture, manufacturing, construction, service, hospitality, and retail industries and other areas of employment.

Overall, 15 674 of 17 005 respondents (92.2%) reported any reactions after their booster or third dose of a COVID-19 vaccine (Table 2). Local reactions at the vaccination site (pain, erythema, and/or swelling) were reported by 14 074 of 17 005 patients (82.8%), while systemic reactions were reported by 11 542 of 17 005 patients (67.9%). The most common symptoms reported were pain at injection site (13 972 of 17 005 [82.2%]) and fatigue (9247 of 17 005 [54.4%]). The mean (SD) maximum temperature among 1544 participants with fever who reported their temperature was 38.3° (0.6°) C after their booster or third vaccine dose. Only 64 of 17 014 participants (0.4%) sought medical care after the booster or third dose. Nearly half (7683 of 16 913 [45.4%]) of participants were at work or planned to go to work on the day of the booster or third dose, and only 5.7% (434 of 7672) either left work or called out of work because of vaccine reactions. Among those who did not work on the day of vaccination, 6327 of 9212 (68.7%) scheduled it on a nonworkday or were on leave. Of those who did work, most (6659 of 7672 [86.8%]) reported minimal or no effect of the vaccine on work. Only 6.3% (1067 of 16 989) reported that the vaccine reactions significantly affected their ability to perform activities of daily living.

**Table 2.** Reported Reactions and Perceptions About Booster or Third Dose of the COVID-19 Vaccine, by Pregnancy and Lactation Status (N = 16 162)

Reaction	Total No.^a^	aOR (95% CI) or difference in mean values (comparing pregnant vs nonpregnant and nonlactating)^b^	*P* value	Total No.^c^	aOR (95% CI) or difference in mean values (comparing lactating vs nonlactating and nonpregnant)^b^	*P* value
Reported	6417			14 238		
At injection site						
Local pain		1.2 (1.1 to 1.4)	.01		1.1 (1.0 to 1.2)	.03
Redness		0.9 (0.7 to 1.1)	.26		1.0 (0.9 to 1.2)	.74
Swelling		0.7 (0.6 to 0.9)	.001		0.9 (0.8 to 1.0)	.05
Myalgias		0.6 (0.5 to 0.7)	<.001		1.0 (1.0 to 1.1)	.36
Fatigue		1.0 (0.9 to 1.1)	.47		1.0 (0.9 to 1.0)	.46
Headache		0.7 (0.7 to 0.8)	<.001		1.1 (1.0 to 1.1)	.17
Chills		0.6 (0.5 to 0.6)	<.001		1.1 (1.0 to 1.2)	.16
Fever		0.5 (0.4 to 0.6)	<.001		1.0 (0.9 to 1.1)	.70
Gastrointestinal symptoms		0.8 (0.6 to 1.0)	.08		1.0 (0.8 to 1.1)	.75
Any local reaction		1.2 (1.0 to 1.4)	.01		1.1 (1.0 to 1.2)	.06
No. of local reactions^d^		0.0 (−0.04 to 0.03)	.81		0.0 (−0.02 to 0.03)	.54
Any systemic reaction		0.7 (0.6 to 0.8)	<.001		1.0 (0.9 to 1.1)	.51
No. of systemic reactions^d^		−0.3 (−0.4 to −0.2)	<.001		0.0 (−0.04 to 0.08)	.47
Sought medical care or advice within 24 h after receiving vaccine booster or third dose	6416	2.3 (1.1 to 4.8)	.03	14 238	1.0 (0.6 to 1.9)	.91
Went to work or planned to go to work on day of booster or third dose	6391	0.9 (0.8 to 1.0)	.23	14 164	0.6 (0.6 to 0.7)	<.001
Vaccine						
Had a significant impact on ability to work on the day of the booster or third dose	3164	0.6 (0.4 to 0.8)	.003	6062	1.0 (0.9 to 1.3)	.71
Had a somewhat or significant impact on ability to perform daily activities	6408	0.8 (0.7 to 1.0)	.007	14 226	0.9 (0.9 to 1.0)	.05
Dose with most severe perceived symptoms^e^	6157			14 649		
First		1.2 (1.0 to 1.4)	.13		1.0 (0.9 to 1.1)	.98
Second		1.3 (1.1 to 1.6)	<.001		1.0 (0.9 to 1.1)	.70
Booster or third		0.7 (0.6 to 0.8)	<.001		1.0 (0.9 to 1.2)	.48
Received influenza vaccine this season (2021-2022)	6056	2.7 (1.7 to 4.3)	<.001	13 278	1.1 (0.9 to 1.4)	.30
Reported any hesitancy to get the booster or third dose	6345	2.3 (2.0 to 2.7)	<.001	14 087	0.8 (0.7 to 0.9)	<.001
Discussed booster or third dose with a health care professional	6396	25.8 (22.3 to 29.8)	<.001	14 183	1.2 (1.1 to 1.3)	<.001
Received a recommendation to get the booster or third dose	5967	6.8 (5.8 to 8.0)	<.001	12 796	1.0 (0.9 to 1.1)	.78
Recommendations from public or medical health authorities were helpful in decision about a booster or third dose	6204	1.2 (1.0 to 1.4)	.06	13 789	1.2 (1.0 to 1.3)	.01

Abbreviation: aOR, adjusted odds ratio.

^a^ Comparing cohort of pregnant individuals with cohort of individuals who were neither pregnant nor lactating (reference). The number includes the total number of individuals who were pregnant and those who were neither pregnant nor lactating included in each comparison.

^b^ The odds ratios from logistic regression models and the differences in mean values from linear regression models were adjusted for vaccine type, age, race, area of employment, educational level, parity, and number of days elapsed from receiving vaccine dose until filling out the survey. The measure of association is the difference in mean values for the number of local reactions and the number of systemic reactions.

^c^ Comparing cohort of lactating individuals with cohort of individuals who were neither pregnant nor lactating (reference). The number includes the total individuals who were lactating and those who were neither pregnant nor lactating included in each comparison.

^d^ The measure of association is the difference in mean values for the number of local reactions and the number of systemic reactions.

^e^ Among individuals who received BNT162b2 or mRNA-1273 for their primary vaccine series. The reference category was “similar reactions or symptoms for all doses.”

**Figure 1.**
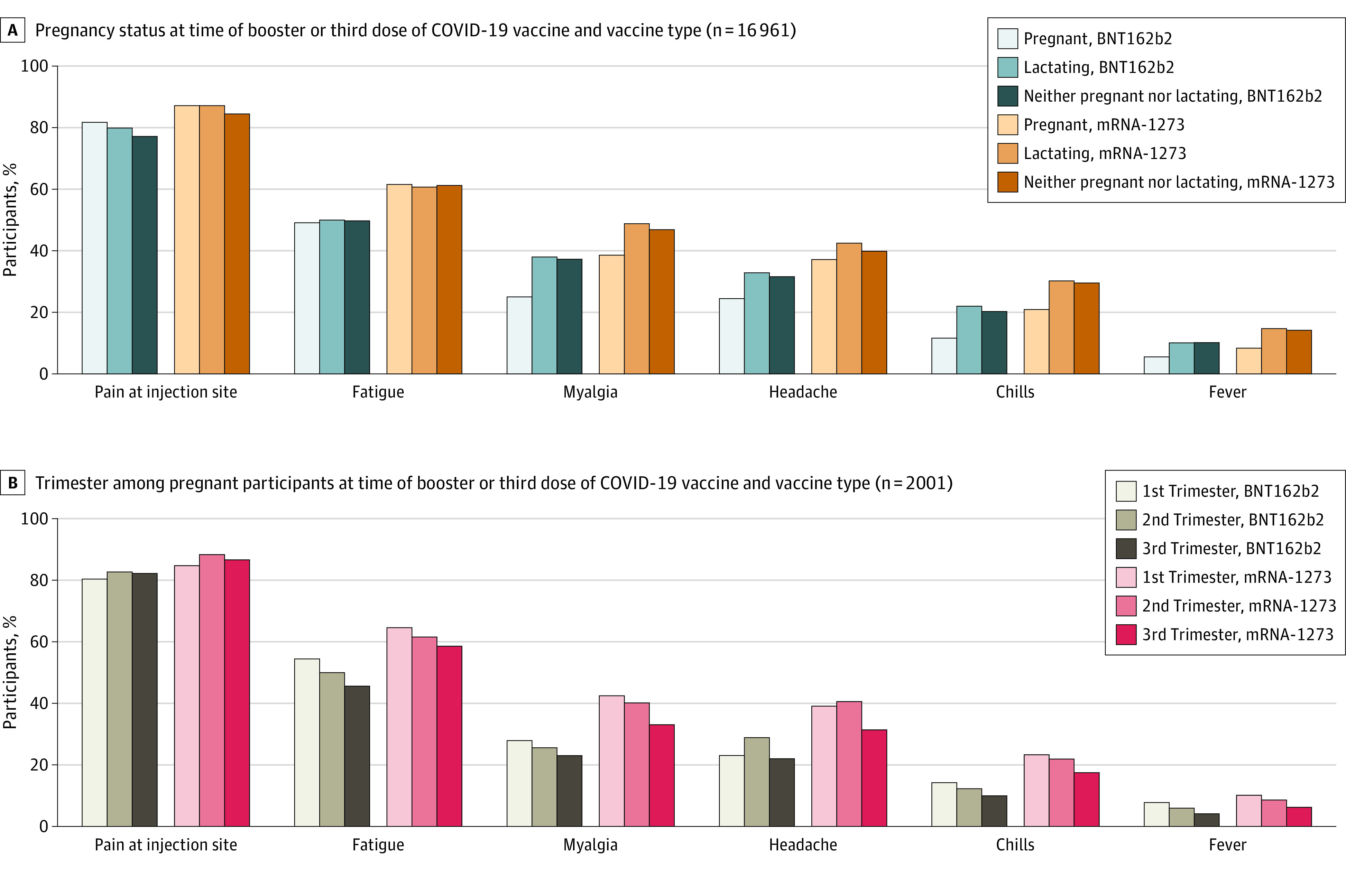
Short-term Local and Systemic Reactions to the Booster or Third Dose of a COVID-19 Vaccine by Pregnancy Status and Vaccine Type and by Trimester

Compared with individuals who were lactating and those who were neither pregnant nor lactating, pregnant individuals reported similar rates of local reactions but reported fewer systemic symptoms after vaccination (Figure 1A). Pregnant participants in their third trimester at the time of the booster or third COVID-19 vaccine dose reported fewer systemic symptoms than pregnant participants in their first and second trimesters (Figure 1B). Compared with individuals who were neither pregnant nor lactating, pregnant participants were more likely to report pain at the injection site (adjusted odds ratio [aOR], 1.2; 95% CI, 1.1-1.4), swelling at injection site (aOR, 0.7; 95% CI, 0.6-0.9), or systemic symptoms, such as myalgias (aOR, 0.6; 95% CI, 0.5-0.7), headache (aOR, 0.7; 95% CI, 0.7-0.8), chills (aOR, 0.6; 95% CI, 0.5-0.6), fever (aOR, 0.5; 95% CI, 0.4-0.6), and any local reaction to a COVID-19 booster or third dose (aOR, 1.2; 95% CI, 1.0-1.4) but were less likely to report any systemic reaction (aOR, 0.7; 95% CI, 0.6-0.8) (Table 2). Individuals who were lactating and those who were neither pregnant nor lactating reported similar reactions after the booster or third dose. Among pregnant participants, only 48 (2.4%) reported any obstetrical symptoms after their vaccination. The most commonly reported obstetrics-related symptom within 24 hours of receipt of a COVID-19 booster or third dose was contractions (11 of 2009 [0.6%]), which were reported only by participants in their third trimester. Among lactating individuals, 10 243 of 10 278 (99.7%) did not interrupt breastfeeding for their booster or third dose, 83 of 10 278 (0.8%) reported an increase in breast milk supply, 355 of 10 278 (3.5%) reported a decrease in breast milk supply, and 121 of 10 278 (1.2%) reported any issues with their breastmilk-fed infant after vaccination.

We evaluated the experiences of participants with vaccine doses and reactogenicity; 7408 of 16 270 participants (45.5%) who received an mRNA-based vaccines for their primary vaccine course reported the most severe reactions with the second COVID-19 vaccine dose, while 4273 of 16 270 (26.3%) reported the most severe reactions with the booster or third dose. Pregnant participants were less likely to report the most severe symptoms with the booster or third dose compared with participants who were neither pregnant nor lactating at the time of their booster or third dose (aOR, 0.7; 95% CI, 0.6-0.8; *P* < .001), while there was no difference between lactating individuals and individuals who were neither pregnant nor lactating at the time of their booster or third dose in reporting the most severe symptoms with the booster or third dose (Table 2). Most participants (15 443 of 16 954 [91.1%]) received the seasonal influenza vaccine, including 1905 of 2005 pregnant participants (95.0%). Pregnant participants were more likely to report that they received an influenza vaccine compared with participants who were neither pregnant nor lactating at the time of their COVID-19 booster or third dose (aOR, 2.7; 95% CI, 1.7-4.3; *P* < .001). There was no difference in the receipt of influenza vaccine between lactating individuals and individuals who were neither pregnant nor lactating.

**Figure 2.**
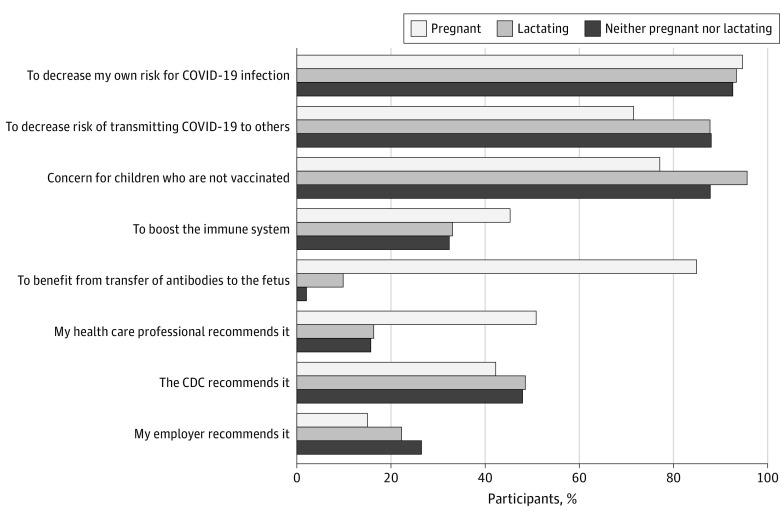
Reasons for Receiving a Booster or Third Dose of a COVID-19 Vaccine by Pregnancy Status at the Time of Booster or Third Dose Vaccination (N = 16 976) Answer choices were not mutually exclusive. CDC indicates Centers for Disease Control and Prevention.

Pregnant participants were significantly more likely to report any hesitancy in receiving the COVID-19 vaccine booster or third dose compared with individuals who were neither pregnant nor lactating (aOR, 2.3; 95% CI, 2.0-2.7; *P* < .001) (Table 2). However, pregnant participants were also significantly more likely to report that they discussed the COVID-19 booster or third dose with a health care professional compared with individuals who were neither pregnant nor lactating (aOR, 25.8; 95% CI, 22.3-29.8; *P* < .001) and to report that they received a recommendation to receive the dose (aOR, 6.8; 95% CI, 5.8-8.0; *P* < .001). Factors that were associated with receipt of a booster or third vaccine dose included the desire to decrease one’s own risk for COVID-19 infection (15 835 of 16 976 [93.3%]), to decrease the risk of transmitting COVID-19 infection to others (14 580 of 16 976 [85.9%]), concern for children who are unvaccinated (15 489 of 16 976 [91.2%]), and the potential transfer of increased concentration of antibodies to the fetus (among pregnant participants) (1706 of 2007 [85.0%]) (Figure 2).

**Figure 3.**
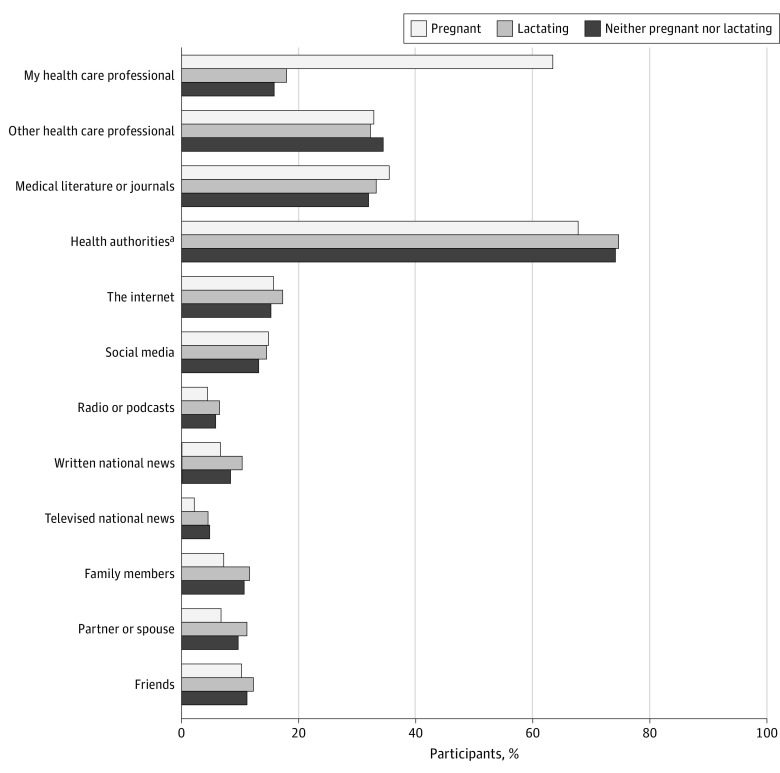
Information Sources for the COVID-19 Vaccine Booster or Third Dose by Pregnancy Status at the Time of Booster or Third Dose Vaccination (N = 16 710) Answer choices were not mutually exclusive. ^a^Examples of health authorities included were the Centers for Disease Control and Prevention, state department of health, American Medical Association, and American College of Obstetricians and Gynecologists.

All 3 cohorts reported that recommendations from public or medical health authorities were helpful in deciding about receiving a COVID-19 vaccine booster or third dose (1744 of 1938 individuals who were pregnant [90.0%], 8946 of 9948 individuals who were lactating [89.9%], and 4028 of 4570 individuals who were neither pregnant nor lactating [88.1%]; *P =* .004). Pregnant participants were significantly more likely to list their health care professional as an important source for information about the COVID-19 booster or third dose (1263 of 1991 [63.4%]) compared with individuals who were lactating (1811 of 10 114 [17.9%]) or who were neither pregnant nor lactating (730 of 4605 [15.9%]; *P* < .001). Other important sources of information were health authorities, such as the Centers for Disease Control and Prevention, public health departments, and the American College of Obstetricians and Gynecologists (12 319 of 16 710 [73.7%]); other health care professionals (5503 of 16 710 [32.9%]); and medical literature or journals (5548 of 16 710 [33.2%]) (Figure 3).

## Discussion

This large prospective cohort study found that the COVID-19 vaccine booster or third dose was well tolerated among individuals who were pregnant, lactating, or neither pregnant nor lactating. The frequencies of local and systemic reactions after the administration of the booster dose among these 3 groups were comparable to those reported in vaccine clinical trials among the general population, as well as national safety surveillance systems specific to booster doses.^4,5,6,7^ We found that the frequency of local reactions to the booster dose was greater than systemic reactions, with pain at the injection site, fatigue, myalgia, and headache as the most common short-term reactions after a COVID-19 vaccine booster dose. The pattern of reactogenicity after a booster or third dose between cohorts in our study based on pregnancy status were similar to those who received the BNT162b2 and those who received mRNA-1273 vaccines, and booster or third dose reactions in our cohort were also similar to second dose reactions previously reported in this same cohort.^8^ Similarly, among pregnant participants, the prevalence of obstetrical symptoms reported after a booster or third dose was lower than the prevalence of obstetrical symptoms reported after a second dose in this cohort.^8^ In our study, pregnant participants frequently reported receiving recommendations for the booster or third dose from their health care professionals, which suggests that clinicians may play a significant role in vaccine acceptance and as a source for vaccine information, a finding supported by previous studies among pregnant individuals.^12,13^ Although general vaccine acceptance was high among this cohort, as evidenced by the high uptake of the influenza vaccine (91.1% of all participants and 95.0% of pregnant participants), the importance of the health care professional’s recommendation is pertinent given the ongoing increased vaccine hesitancy among pregnant individuals in the context of the COVID-19 vaccine.^14^ Uptake of the influenza vaccine among pregnant individuals was substantially higher in our cohort compared with national estimates of the uptake of the influenza vaccine during pregnancy reported in the 2021-2022 season.^15^ However, we found that pregnant participants were twice as likely to report hesitance to receive a COVID-19 booster or third dose, which highlights an important gap in vaccine coverage and concerns that hinder receipt of subsequent doses among pregnant individuals. Efforts to ensure that pregnant individuals are given accurate information that addresses safety, teratogenicity, and other health concerns as well as the health benefits associated with vaccination during pregnancy will be needed to overcome these gaps.

The potential to transfer antibodies to the fetus and reduce risk for COVID-19 infection were commonly cited reasons for receiving a COVID-19 booster by pregnant participants. Compared with individuals who were lactating or neither pregnant nor lactating, pregnant participants were also more likely to report boosting the immune system as an important reason for receiving a COVID-19 booster or third dose. These results suggest that pregnant individuals may be more concerned than nonpregnant individuals about the increased risk of severe illness from COVID-19.^16^

### Strengths and Limitations

The strengths of this study include the ability to compare vaccine reactions and perceptions between individuals who were pregnant or lactating with individuals of similar age and fertility intentions who were neither pregnant nor lactating, the large sample size, and the participation from individuals across all 50 states and several US territories, including Guam, Northern Mariana Islands, Puerto Rico, and US Virgin Islands. In addition, the updated survey allowed us to compare findings within the cohort with data from the primary vaccine series.^8^

Our study is also subject to some limitations. Sampling was via a convenience sample reflecting the early wave of COVID-19 vaccinations, largely composed of health care workers owing to vaccine eligibility at the time of the launch of this ongoing study. The prevalence of vaccine reactions may be biased because they were self-reported. Furthermore, most participants identified as White, which limits the generalizability of the study findings. In addition, analyses of pregnancy outcomes of participants who were pregnant when vaccinated are in progress.

## Conclusions

The findings of this cohort study suggest that COVID-19 boosters or third doses were well tolerated by individuals who were pregnant and lactating. Data on COVID-19 vaccine boosters are particularly important as vaccine uptake during pregnancy is lagging, and strategies to reduce vaccine hesitancy, increase vaccine acceptance, and help guide discussions between pregnant and lactating persons and maternal care professionals are needed.

## Notes

Supplement. **eAppendix.** COVID-19 Vaccine 3rd Dose/Booster Dose Follow-up Questions
