# Supplementary material for: Analysis of Vaccine Reactions After COVID-19 Vaccine Booster Doses Among Pregnant and Lactating Individuals
Source: JAMA Netw Open. 2022 Sep 8;5(9):e2230495. doi: 10.1001/jamanetworkopen.2022.30495 (PMC9459662; doi:10.1001/jamanetworkopen.2022.30495)
Supplement: Supplement. — eAppendix. COVID-19 Vaccine 3rd Dose/Booster Dose Follow-up Questions [file jamanetwopen-e2230495-s001.pdf]

Supplementary Online Content

Kachikis A, Englund JA, Covelli I, et al. Analysis of vaccine reactions after COVID-19 vaccine booster doses among pregnant and lactating individuals. *JAMA Netw Open.* 2022;5(9):e2230495. doi:10.1001/jamanetworkopen.2022.30495

eAppendix. COVID-19 Vaccine 3rd Dose / Booster Dose Follow-up Questions

This supplementary material has been provided by the authors to give readers additional information about their work.

© 2022 Kachikis A et al. *JAMA Network Open.*

eAppendix. COVID-19 Vaccine 3rd Dose / Booster Dose Follow-up Questions

This form contains questions about your experience with the 3rd or booster dose of the COVID-19 vaccine.

If you originally received the J&J/Janssen vaccine, the booster or additional dose will be your second dose.

ALL ANSWERS ARE CONFIDENTIAL.

Date survey was filled out: _________________

What was the date of your 3rd or BOOSTER dose of the COVID-19 vaccination?
____

What was the indication for your 3rd or BOOSTER dose of the COVID-19 vaccine?
- O Immunocompromised status
- O Healthcare worker
- O Essential worker
- O Pregnancy status
- O Underlying health condition
- O Other (Please specify: __________)
- O None of the above

What was the name of the manufacturer of your 3rd/booster dose?
- O Pfizer
- O Moderna
- O Janssen
- O AstraZeneca
- O Sanofi
- O Novavax
- O Other (Please specify: _________)
- O I don't know

Were you pregnant at the time of your 3rd/booster dose?
- O Yes
- O No

© 2022 Kachikis A et al. *JAMA Network Open.*

How many weeks along in pregnancy (gestational age) were you when you received the 3rd/booster dose of the COVID-19 vaccine? (In weeks. Please round down (e.g., 26 weeks 3 days -> 26): ____________________________________

Were you breastfeeding (pumping) at the time of your 3rd/booster dose?
- O Yes
- O No

In the first 24 hours after your 3rd/booster dose, did you experience any of the following? (Select all that apply)
- ▭ Local pain at the site of vaccination
- ▭ Redness at the site of vaccination
- ▭ Swelling at the site of vaccination
- ▭ Muscle aches, myalgia
- ▭ Fatigue
- ▭ Headache
- ▭ Chills
- ▭ Fever
- ▭ GI symptoms (diarrhea, stomach upset, etc.)
- ▭ Severe allergic / anaphylactic reaction with throat swelling
- ▭ Severe allergic reaction with rash / hives
- ▭ Other (Please specify: ______)
- ▭ None of the above

How many hours did your symptom(s) described above last? (in hours. Please type '99' if you prefer not to answer): ____________________

What was the highest temperature you recorded with your fever? (Please enter '9999' if you did not record a number or if you prefer not to answer): ________________________

Fever units
- O Fahrenheit
- O Celsius

How many hours did your fever last? (In hours; Please type '99' if you prefer not to answer): __________________

© 2022 Kachikis A et al. *JAMA Network Open.*

Did you take any medication to treat your fever?

- ○ Yes
- ○ No
- ○ Prefer not to answer

Did you seek medical care or advice within 24 hours after receiving your 3rd/booster COVID-19 vaccine dose?

- ○ Yes
- ○ No
- ○ Prefer not to answer

If you did seek medical care or advice, why? (Select all that apply)

- ☐ Respiratory concern
- ☐ Gastrointestinal concern
- ☐ Skin concern / rash
- ☐ Blood pressure concern
- ☐ Other (Please explain: _________)
- ☐ Prefer not to answer

Were you at work or planning to work after receiving the vaccination on the day of your 3rd/booster dose of the COVID-19 vaccine?

- ○ Yes
- ○ No
- ○ Prefer not to answer

What was the reason you were not working on the day or in the time after you received the 3rd/booster dose of the COVID-19 vaccine?

- ○ I took the day off from work.
- ○ I scheduled the vaccination on a day that I did not have to work.
- ○ I scheduled the vaccination for a time following my shift at work.
- ○ I was on leave.
- ○ Not applicable
- ○ Prefer not to answer

Did the vaccine (and any potential side effects) impact your work on the day of your 3rd/booster dose of the COVID-19 vaccine?

- ○ Yes. I did not go to work or I had to leave work after the vaccination.
- ○ Yes. The side effects from the vaccine significantly affected my work, but I did not have to leave work.
- ○ Yes, but the side effects were minimal
- ○ No, not at all
- ○ Prefer not to answer

© 2022 Kachikis A et al. *JAMA Network Open.*

Did you take any days off of work after your 3rd/booster dose of the COVID-19 vaccine (not including the day you got the vaccination) because of side effects that you thought were due to the vaccine?

- Yes
- No
- Not applicable
- Prefer not to answer

If so, how many days did you have to take off? (in days. Please type '99' if you prefer not to answer): ________________

Did the vaccine (and any potential side effects) impact your ability to perform your normal daily activities (i.e., cooking, showering, childcare, etc.)?

- Yes. The side effects from the vaccine significantly affected my ability to perform daily activities.
- Yes. The side effects from the vaccine somewhat affected my ability to perform daily activities.
- No. The side effects from the vaccine were minimal.
- No. I did not experience any effects from the vaccine.
- Prefer not to answer

Please add any comments on the 3rd booster dose of the vaccine's effect on your work or life activities: (Please type '99' if you prefer not to answer): ______________________________

[If pregnant] In the first 24 hours after receiving the vaccine, did you have any of the following? (Select all that apply)

- ☐ Contractions or labor
- ☐ Breaking of your baby's bag of water
- ☐ Low fluid around the baby
- ☐ Concerning baby heart monitoring
- ☐ Concerns about the baby's movement
- ☐ Vaginal bleeding
- ☐ Higher blood pressures
- ☐ Higher blood sugar levels
- ☐ More vaginal discharge
- ☐ Other (Please explain: ________)
- ☐ None of the above

© 2022 Kachikis A et al. *JAMA Network Open.*

Since receiving the 2nd dose of the vaccine, have you experienced any other pregnancy concerns that you believe are due to the vaccine?

- Yes
- No

If yes, which vaccine-related pregnancy effects have you experienced? (Please type 'N/A' if you prefer not to answer) ________________________________

[If lactating] Did you interrupt breastfeeding for the 3rd/booster dose of the COVID-19 vaccine?

- Yes, stopped for a short time
- Yes, stopped altogether
- No
- Prefer not to answer

If yes, how long did you stop breastfeeding for? (In hours. Please type '9999' if you prefer not to answer) ______________________

Did you notice any change in your breast milk supply after receiving the COVID-19 vaccine?

- Yes, an increase in breast milk supply
- Yes, a decrease in breast milk supply
- No
- Prefer not to answer

How long did the change in breast milk supply last? (Please type '99' if you prefer not to answer.) ______________________________________

How much of a change in breast milk supply did you notice? (Approximate amount in mL)

____

Since receiving the 3rd/booster dose of the COVID-19 vaccine, have you noticed any concerns in your infant after breastfeeding?

- Yes
- No
- Prefer not to answer

If concerns in your infant after breastfeeding, please explain: (Please type '99' if you prefer not to answer) _______________________________

© 2022 Kachikis A et al. *JAMA Network Open.*

This section asks some final questions about your 3rd/booster dose of the COVID-19 vaccine.

If you compared your symptoms or reactions after the 2nd dose of the COVID-19 vaccine compared to the 1st dose, which of the following statements would you agree with?

- I experienced more/worse reactions with the 2nd dose of the vaccine, compared to the 1st.
- My symptoms/reactions with the 1st and 2nd doses of the vaccine were about the same.
- I experienced fewer/milder reactions with the 2nd dose of the vaccine, compared to the 1st.
- Not applicable - received a one-dose vaccine as primary COVID-19 vaccine series

If you compared your symptoms or reactions after the 3rd/booster dose of the COVID- 19 vaccine compared to the 2nd dose, which of the following statements would you agree with?

- I experienced more/worse reactions with the 3rd/booster dose of the vaccine, compared to the 2nd.
- My symptoms/reactions with the 2nd and 3rd/booster doses of the vaccine were about the same.
- I experienced fewer/milder reactions with the 3rd/booster dose of the vaccine, compared to the 2nd.
- Not applicable - received a one-dose vaccine as primary COVID-19 vaccine series

With which dose of the COVID-19 vaccine did you experience the most symptoms/reactions?

- the 1st dose
- the 2nd dose
- the 3rd dose/booster dose
- I had similar reactions / symptoms with all doses
- I'm not sure

Did you discuss the 3rd dose / booster dose with a healthcare provider?

- Yes, with my OB healthcare provider
- Yes, with another healthcare provider
- No
- Don't know
- Prefer not to answer

Was the 3rd dose / booster dose of the vaccine recommended to you?

- Yes
- No
- Don't know

Prefer not to answer.

© 2022 Kachikis A et al. *JAMA Network Open.*

If yes, who recommended the 3rd dose / booster dose?

- My OB healthcare provider
- Other healthcare provider
- A co-worker
- A family member
- Other (Please specify: _______)
- Prefer not to answer

Have you received the flu shot for this season?

- Yes
- Not yet but plan to get
- No
- Not sure

Did you receive the flu shot at the same time/same vaccination session as the 3rd vaccine dose/ booster dose?

- Yes
- No

If you compared your symptoms or reactions after the flu shot compared with the 3rd/booster dose of the COVID-19 vaccine, which of the following statements would you agree with?

- I experienced more/worse reactions with the 3rd/booster dose of the vaccine, compared to the flu shot.
- My symptoms/reactions with the 3rd/booster dose of the vaccine and the flu shot were about the same.
- I experienced fewer/milder reactions with the 3rd/booster dose of the vaccine, compared to the flu shot.

© 2022 Kachikis A et al. *JAMA Network Open.*

Where did you get most of your information about the 3rd dose / booster dose?

- ▭ Your doctor
- ▭ Other health professionals
- ▭ Medical literature/journals
- ▭ Health authorities (e.g. CDC, state, department of health, AMA, ACOG, etc)
- ▭ Complementary, alternative or naturopathic health providers
- ▭ The internet (e.g. web search, webMD, etc)
- ▭ Social media (e.g. Facebook, Twitter, Instagram, TikTok, etc.)
- ▭ Online message boards, reddit, blogs
- ▭ Radio / podcasts (e.g. NPR, Ted talks, etc)
- ▭ Written local news (e.g. local newspaper)
- ▭ Written national news (e.g. national newspapers or services)
- ▭ Televised local news
- ▭ Televised national news (e.g. CNN, Fox News, MSNBC, etc)
- ▭ Religious leaders
- ▭ Political leaders
- ▭ Your employer
- ▭ Your family members
- ▭ Your partner
- ▭ Friends
- ▭ Teachers
- ▭ Other (Please specify: ________)
- ▭ None of the above

Were recommendations from public health groups / authorities (for example, the CDC) important in helping you make the decision to get a 3rd dose / booster dose?

- O Yes
- O Don't know
- O No

Of public health departments or national health authorities, which recommendations for the 3rd dose/ booster dose were particularly important to you?

- ▭ CDC (Centers for Disease Control and Prevention
- ▭ DOH (Your State Department of Health)
- ▭ NIH (National Institutes of Health)
- ▭ ACOG (American College of Obstetricians and Gynecologists)
- ▭ RCOG (Royal College of Obstetricians and Gynaecologists)
- ▭ SMFM (Society for Maternal-Fetal Medicine)
- ▭ Other (Please specify: ________)
- ▭ None of the above

© 2022 Kachikis A et al. *JAMA Network Open.*

Were you at all hesitant to get the 3rd dose / booster dose of the COVID-19 vaccine?

- o Yes
- o Don't know
- o No

What were your motivations for getting a 3rd dose / booster dose of the COVID-19 vaccine? (Please select any that apply)

- ☐ To decrease my risk of getting a breakthrough COVID-19 infection.
- ☐ To decrease the risk of transmitting COVID-19 to my family and friends.
- ☐ Out of concern for my child or children who are not vaccinated.
- ☐ The CDC recommended it.
- ☐ My doctor/health provider recommended it.
- ☐ My employer recommended it.
- ☐ To benefit from transfer of my antibodies to my fetus.
- ☐ To boost my immune system.
- ☐ It is a recommended dose. Otherwise, no specific reason.
- ☐ Other (Please specify: ________)
- ☐ None of the above

Are there any other comments/concerns about receiving the 3rd/booster dose of the vaccine that you would like us to know about? (Please type '99' if you prefer not to answer)

____

© 2022 Kachikis A et al. *JAMA Network Open.*
